# Assessment of the Potential of Electrochemical Steps in Direct Air Capture through Techno-Economic Analysis

**DOI:** 10.1021/acs.energyfuels.4c02202

**Published:** 2024-08-06

**Authors:** Natalie Rosen, Andreas Welter, Martin Schwankl, Nicolas Plumeré, Júnior Staudt, Jakob Burger

**Affiliations:** † Laboratory of Chemical Process Engineering, Technical University of Munich, Campus Straubing for Biotechnology and Sustainability, 94315 Straubing, Germany; ‡ BMW Group, 85748 Garching, Germany; § Professorship for Electrobiotechnology, Technical University of Munich, Campus Straubing for Biotechnology and Sustainability, 94315 Straubing, Germany

## Abstract

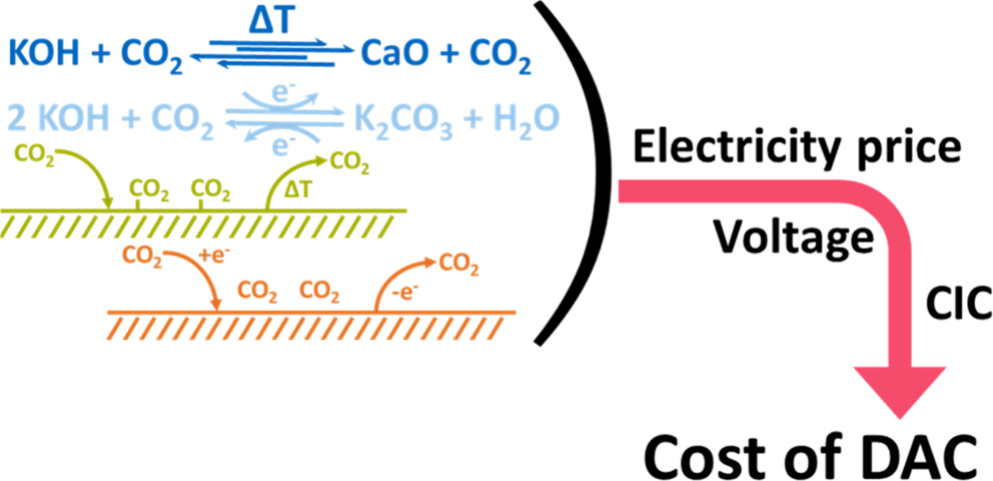

Direct air capture (DAC) technologies are proposed to reduce the atmospheric CO_2_ concentration to mitigate climate change and simultaneously provide carbon as a feedstock independent of fossil resources. The currently high energy demand and cost of DAC technologies are challenging and could limit the significance of DAC processes. The present work estimates the potential energy demand and the levelized cost of capture (LCOC) of liquid solvent absorption and solid adsorption DAC processes in the long term. A consistent framework is applied to compare nonelectrochemical to electrochemical DAC processes and estimate the LCOC depending on the electricity price. We determine the equivalent cell voltage needed for the electrochemical steps to achieve comparable or lower energy demand than nonelectrochemical processes. The capital expenses (CapEx) of the electrochemical steps are estimated using analogies to processes that are similar in function. The results are calculated for a range of initial data of CapEx and energy demand to include uncertainties in the data.

## Introduction

### Direct Air Capture Technologies

**Figure 1:**
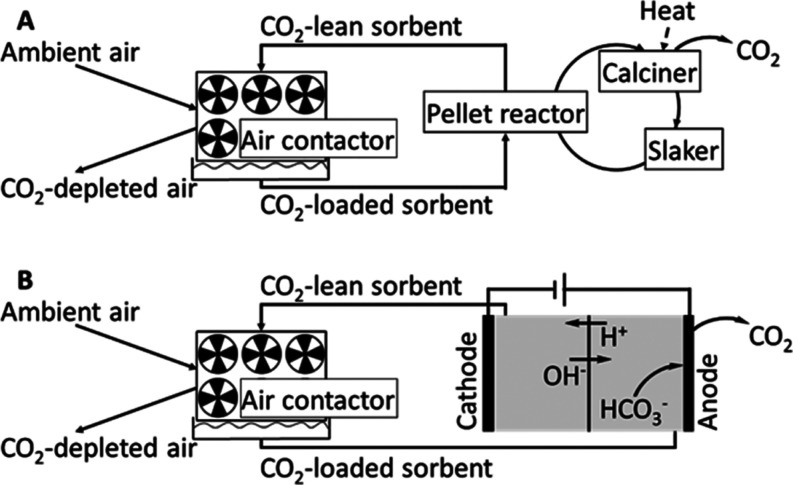
Schematic of absorption-based processes. (A) Absorption occurred *via* chemical looping and (B) absorption with electrochemical regeneration.

Negative emission technologies (NETs), designed to reduce the concentration of greenhouse gases in the atmosphere,^1−4^ are needed to achieve the target of the 2015 Paris Agreement of restricting global warming to “well below 2 °C above pre-industrial levels”.^5^ These technologies are necessary for offsetting the emissions of hard-to-abate industries. The National Academy of Sciences considers six approaches for NETs, including bioenergy with carbon capture and sequestration (BECCS), carbon mineralization, and direct air capture (DAC), and classifies their application potential based on societal, environmental, and economic impacts.^6^ In addition to reducing greenhouse gases, the recovery of carbon adds further incentive to the application potential of these technologies as carbon is a valuable element essential for the chemical industry and the production of fuels. In a future carbon cycle without abundant fossil resources, securing carbon feedstock necessitates chemical recycling, the use of biomass, postcombustion capture (PCC), and DAC.^7^ The latter is scalable and independent of location and time, which means that CO_2_ emitted in the past can be captured and used as a carbon feedstock today. The different DAC technologies have various characteristics and challenges. In particular, the high energy demand and costs are the bottlenecks of current technologies.^6^ To be considered economical, the National Academy of Science^6^ argued in 2019 that the cost of NETs should not exceed a limit of 100 $/t_CO2_ for the Levelized cost of capture (LCOC). Currently, the DAC cost is a factor of 5–6 above this limit, depending on the process design.^8^ Therefore, a significant cost reduction for DAC processes is necessary to make them economical. Lackner and Azarabadi^9^ analyzed the constraints for DAC cost reduction, and their buy-down model showed that investment and an increase in the annual capture capacity could reduce DAC costs. Lackner also introduced the first concept for DAC in 1999, making use of the exothermic reaction between calcium hydroxide and carbon dioxide.^10^ This laid the foundation for DAC *via* absorption using alkali and alkaline earth hydroxides, from which the absorption process *via* chemical recovery loops emerged (see Figure 1A).^11,12^ In the first loop, the capture loop, air is fed through a contactor, where the CO_2_ is absorbed in an aqueous alkali hydroxide solution, forming soluble carbonates. The loaded solvent is regenerated in the pellet reactor using calcium hydroxide to obtain calcium carbonate. The second loop, the regeneration loop, aims to recover CO_2_ from calcium carbonate by heating it above 900 °C in the calciner. The highly endothermic reaction produces calcium oxide, which is slaked at 300 °C with water to yield calcium hydroxide and close the loop.^13^ Although absorption *via* chemical looping (ACL) is already a far-developed process, some potential improvements regarding DAC aspects are mentioned in the literature. For example, González et al.^14^ suggest improving the sorbent performance of CaO by doping with seawater to minimize sorbent reactivity losses. There is also potential in reactor design and conditions to achieve better performance of the absorption and regeneration steps.^15−17^ Nevertheless, the bottleneck of ACL is the high temperature of ∼900 °C, which requires expensive thermal energy for regeneration. Alternatives to ACL include absorption methods utilizing amines,^18^ amino acids,^18^ and their salts.^19,20^ Amines are common solvents for flue gas capture, but their volatility leads to significant solvent loss through evaporation during the absorption process in DAC.^18^ While amino acids and amino acid salts possess high potential for DAC application, more research is needed, particularly regarding their cyclic capacities and long-term stability.^18^ Additionally, the degradation of amino acids at high temperatures poses a challenge that requires further investigation.^19^ In addition to thermal regeneration, absorption with electrochemical regeneration (AEC)^21^ provides an alternative approach for regenerating the solvent, while the CO_2_ absorption step in the air contactor is similar to ACL (see Figure 1B). Likewise, solvents containing hydroxides and amines can be used for AEC. The regeneration of the CO_2_-loaded sorbent is performed in an electrochemical cell, eliminating the need for high-temperature heat during regeneration. Several approaches exist for designing the electrochemical regeneration cell depending on the sorbent. Saline solutions can be regenerated *via* pH swings, e.g., by bipolar membrane electrodialysis (BPMED).^21^ Thereby, the CO_2_-loaded solution is separated into an acid and a base in an electric field. Carbonates are transported to the anode, where CO_2_ is released.^21^ If amines are used as a sorbent, they can be regenerated *via* electrochemically mediated complexation separation (EMCS).^22^ The amines with bound CO_2_ are fed into an electrochemical cell. Metal ions (e.g., Cu^2+^) are generated through the oxidation of an anode made of the corresponding metal (e.g., copper). The free metal ions form complexes with the amines, which releases the CO_2_. The amines are subsequently regenerated by the reduction of the metal ion at the cathode.^23^ The currently reported differential cell voltages vary for different setups and underlying chemical systems between 0.4 and 1.0 V (for a stoichiometry of one electron transferred per CO_2_ molecule).^24−27^

**Figure 2:**
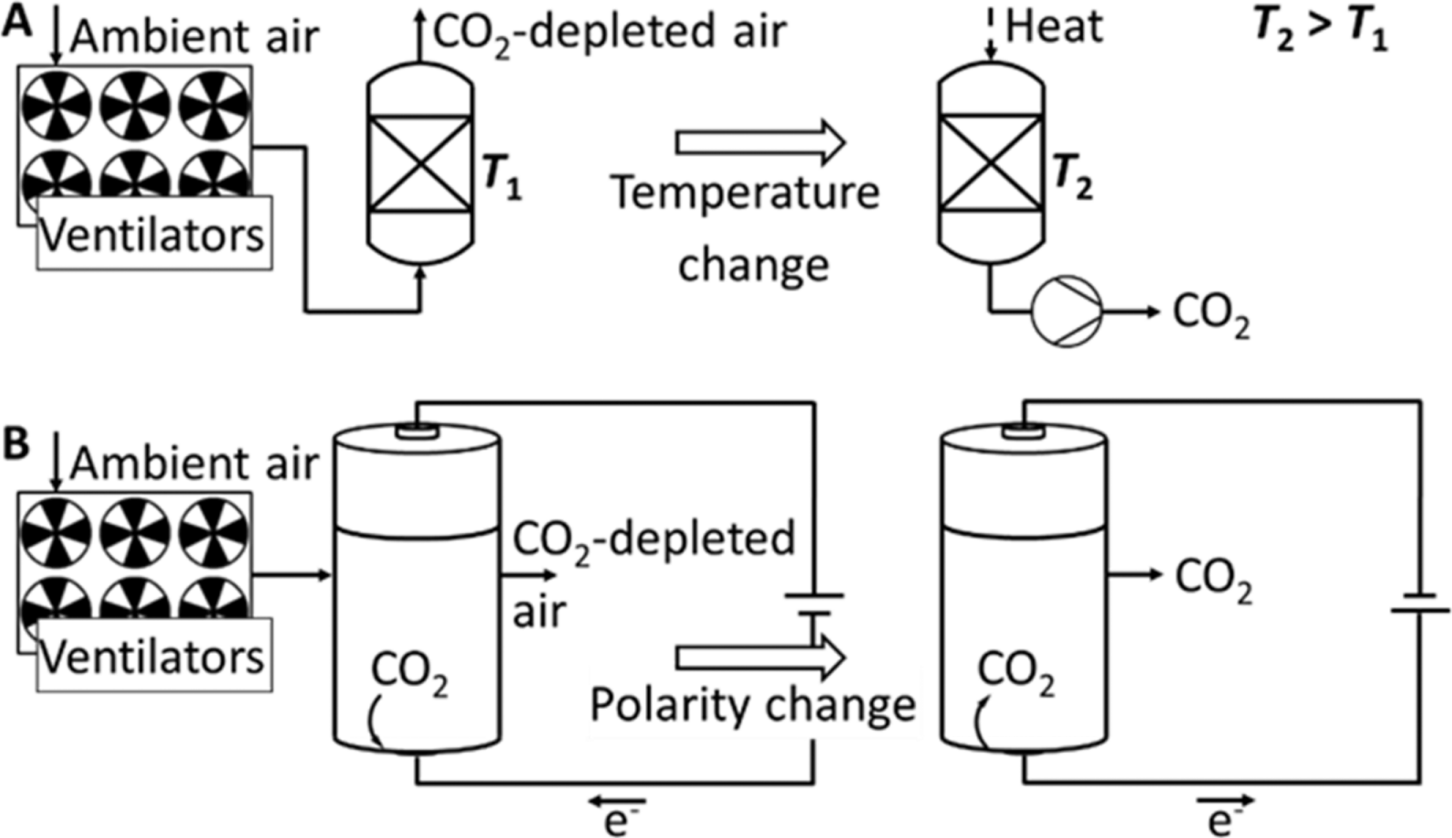
Schematic of adsorption-based processes. (A) Temperature-vacuum swing adsorption and (B) electro-swing adsorption.

The second major class of DAC processes uses adsorption on solids. Figure 2A illustrates temperature-vacuum swing adsorption (TVSA). As a rule, the process is discontinuous, switching between several phases. In phase 1, ambient air is fed through an adsorption unit, where CO_2_ selectively adsorbs to a solid sorbent material at ambient temperature (*T*_1_). When the sorbent is saturated and CO_2_ breaks through, phase 2 starts. The airflow is stopped, and the adsorption bed is heated to the elevated desorption temperature (*T*_2_) at which the CO_2_ desorbs from the surface.^28^ Besides temperature, the thermodynamic driving force in adsorption processes can be shifted by a change in moisture, pressure, electrochemical potential, or a combination thereof.^8^ The sorbent material and the energy demand needed for its regeneration^29^ are the major cost factors of the overall capture cost of TVSA. Improving properties like the capacity^30^ and selectivity of CO_2_ uptake,^31^ the stability,^32^ and a small mass ratio of the contactor material to the adsorbent^31^ lead to less sorbent demand and less sorbent consumption and therefore to optimized costs. Furthermore, the kinetics^8^ and the heat transfer^33,34^ of the adsorption and desorption processes can be optimized. In electro-swing adsorption (ESA), the thermodynamic driving force is influenced by changing the applied voltage (see Figure 2B).^35^ The solid sorbent comprises redox-active moieties such as quinones immobilized on the electrode material.^36^ In the first phase (charging), the quinone-coated electrodes are reduced electrochemically, leading to the adsorption of the CO_2_ in the feed gas stream.^35^ In the second phase (discharging), the quinone moieties are electrochemically oxidized, which releases the CO_2_. A ferrocene-containing electrode serves as an electron source/sink.^35^ Respective reported differential cell voltages range from 1.0 to 2.0 V.^36−38^ Due to oxygen instability, an application with air is not yet possible.^37,39^

Electrochemical processes are often called “promising”^40,41^ or “energy-efficient”.^21,42,43^ In the present work, we set out to verify these claims and estimate the potential energy demand and LCOC of the electrochemical DAC processes (AEC and ESA) in comparison to the nonelectrochemical DAC processes (ACL and TVSA) for different electricity prices. We determine the equivalent cell voltage needed for the electrochemical steps to achieve comparable or lower energy demand than nonelectrochemical processes. The results are calculated for a range of initial data of CapEx and energy demand to include uncertainties of the data.

### Review of Techno-Economic Studies

**Figure 3:**
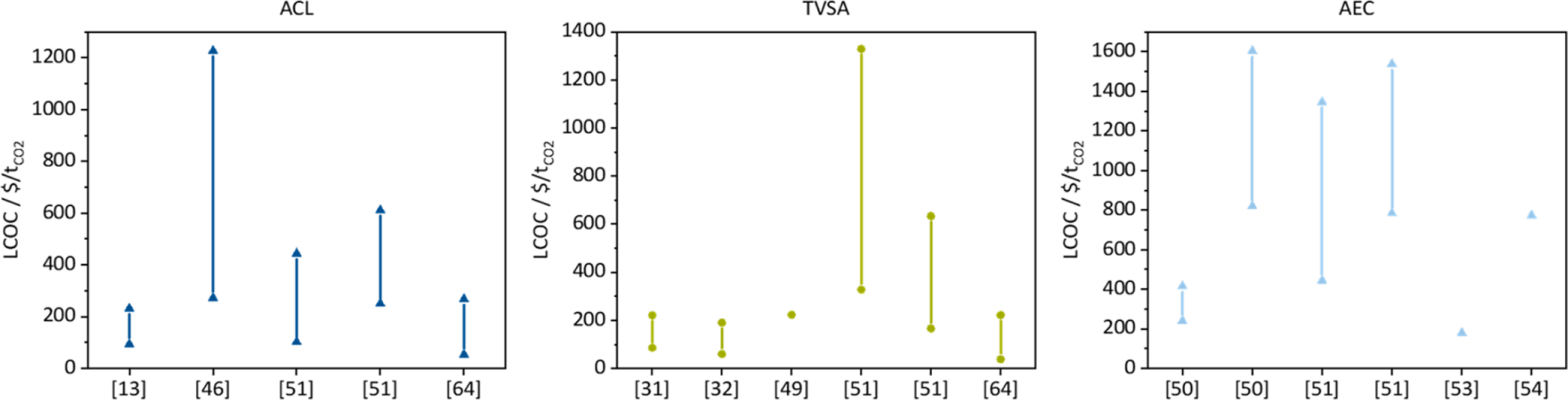
Overview of the levelized cost of capture for absorption with chemical looping (ACL), absorption with electrochemical regeneration (AEC), and temperature-vacuum swing adsorption (TVSA) from different sources (summary of values and references in Table S4).

There are many techno-economic studies on DAC in the literature, which typically report the LCOC. Figure 3 summarizes the reported LCOCs. ACL^13,44−46^ and TVSA^31,32,47−49^ processes are well studied. There are also multiple studies for AEC.^50,51^ There is no LCOC reported for the ESA process yet, to the best of our knowledge. The reported LCOC has a significant variation (more than 1000 $/t_CO_2__) for the individual technologies due to differing assumptions, e.g., on energy cost, plant size, and differently assumed maturity of the processes and involved materials. If more than one value is reported in one study, then different scenarios and conditions are considered. The LCOC for AEC is, on average, higher than for ACL and TVSA. No study predicts costs below 100 $/t_CO_2__ for AEC. For ACL and TVSA, an LCOC below 100 $/t_CO_2__ was mainly estimated for cumulative installed capacities (CICs) > 1 Mt_CO_2__/a.

For ACL, a detailed process design and cost analysis of an air contactor using an alkali hydroxide absorbing solution is reported by Heidel et al.^44^ They propose an intermittently wetted contactor design with cross-flow slab geometry to reduce the capture cost up to 49–80 $/t_CO_2__.^44^ Holmes et al. analyzed the importance of design choices for optimizing the contactor and reported a 4-fold discrepancy between different estimates.^45^ Here, the cost for the contactor design amounts to around 60 $/t_CO_2__.^45^ An energy demand of 2.45 MWh/t_CO2_ natural gas or 1.46 MWh/t_CO2_ natural gas and 0.37 MWh/t_CO_2__ electrical energy is calculated for an entire plant design to provide 1 Mt_CO2_/a, including the capture of CO_2_ and the regeneration of the solvent.^13^ The authors estimate a respective LCOC of 94–232 $/t_CO2_.^13^ The cost range is explained by different energy costs, economic assumptions, and choices in input and output parameters. The influence of these parameters is a crucial aspect in the techno-economic assessment of a conventional liquid-based absorption process using amines.^46^ A cost range of 273–1,227 $/t_CO2_ is reported for the plant design to provide 291 kg_CO2_/h, estimating a thermal energy demand of 2.97 MWh/t_CO2_ and 1.4 MWh/t_CO2_ of electrical energy. The authors report the importance of an innovative gas–liquid contactor in reducing the LCOC.^46^

For TVSA, the total energy demand is estimated to be 0.78 GJ/t_CO2_ of electric and 5.96 GJ/t_CO2_ of thermal energy by Kulkarni and Sholl.^47^ As a sorbent, they consider an amino-modified silica and a structured monolith contactor unit. Using a preliminary process model, they estimated the operational costs to be 95 $/t_CO2_ (it includes the cost for compression of the product).^47^ Sinha and co-workers^32^ performed a modeling study of a TVSA process using metal–organic frameworks (MOFs) as a sorbent material. They identify the sorbent purchase cost, lifetime, and cycle parameters most sensitive to an LCOC of 60–190 $/t_CO2_. The minimum estimated electric and thermal requirement is around 2.3 GJ/t_CO2_ and 0.3 GJ/t_CO2_, respectively.^32^ Process simulations have been used by Sinha et al.^31^ to analyze different scenarios for TVSA with MOFs, from best to worst case. Their results are not based on pilot plant data, so a wide range of performance is possible. For a midrange scenario, their modeling results in electric and thermal energy demands of 0.55–1.12 GJ/t_CO2_ and 3.4–4.8 GJ/t_CO2_, respectively, to result in an LCOC between 86 and 221 $/t_CO2_.^31^ A carbon dioxide removal cost of 100–300 $/t_CO2_ is reported by Climeworks for their long-term target at gigaton scale with the range i.e., caused by unknown factors and boundary conditions, as the electricity cost and cost of labor.^52^ McQueen et al.^49^ performed a cost analysis using a functionalized sorbent and monolith for a 100 ktCO2/a plant capacity. Different energy sources were introduced to calculate the cost of delivering one ton of compressed CO_2_, including all related CO_2_ emissions. Based on a thermal and electric energy demand of 6 GJ/t_CO2_ and 1.5 GJ/t_CO2_, respectively, the total cost of capture amounts to 223 $/t_CO2_ for the base case.^49^ Leonzio et al.^48^ use a mathematical model describing adsorption and desorption stages for two amine-functionalized chemisorbents and three MOFs as physisorbents. They identified the equilibrium loading as a main characteristic influencing the energy consumption and adsorption capacity of the sorbent. The chemisorbents have a corresponding value around 10 times higher than the physisorbents.^48^

For absorption of CO_2_ from flue gas with subsequent BPMED, Iizuka et al.^53^ analyzed the effect of sodium concentration, CO_2_ absorption, recovery ratio, current density, type of membrane, cell numbers, and the flow rate on power consumption and current efficiency. Based on the experimental data, optimal operating conditions were defined, and the respective CO_2_ recovery cost was calculated to be 180 $/t_CO2_ using an electricity cost of 0.12 $/kWh. Improving the current efficiency and reducing the membrane cost could achieve a significant cost reduction, as shown in their work.^53^ Sabatino et al.^54^ developed a cost analysis for DAC using BPMED for solvent regeneration. Based on experimental data of other groups^21,53,55,56^ and an electricity price of 0.06 $/kWh, an energy demand of 1.49 MWh/t_CO2_ and an LCOC of 773 $/t_CO2_ were estimated.^54^ A sensitivity analysis indicated that the high membrane costs make the process expensive.^54^ Sabatino et al.^50^ extended their work with modeling and multiobjective optimization of the BPMED process. They differentiated between a cation-exchange membrane (CEM) and an anion-exchange membrane configuration (AEM). With an electricity price of 0.06 $/kWh and membrane costs of 750 $/m^2^ for BPM and 75 $/m^2^ for ionic exchange membranes, an LCOC of 819–861 $/t_CO2_ (CEM process) and 1420–1604 $/t_CO2_ (AEM process) were reported. For different cases, varying the membrane price, cell resistance, and conductivity of electrolyte solutions, an LCOC was calculated. For a predicted future scenario, where membrane price and resistance were reduced by a factor of 10 compared to the initial cost and an increased conductivity is assumed, the energy demand could be reduced to 17 MJ/kg_CO2_, and an LCOC of 241–272 $/t_CO2_ (CEM process) and 407–415 $/t_CO2_ (AEM process) were indicated.^50^

Although a large number of economic analyses on DAC have already been performed, most of them are on nonelectrochemical processes or discuss single technologies. Comparing the results of different techno-economic analyses is often misleading because the assumptions on boundary conditions strongly influence the results of different studies.^57^ Most techno-economic studies refer to similar literature data and use recalculated values.^58^ Thus, comparing different processes in a consistent framework is interesting.

### Assessment and Comparison of Immature Technologies

A challenge arises when comparing DAC technologies due to the different TRLs. Some processes are only demonstrated on a lab-scale, and others are already in industrial operation with lower LCOC.

The decrease in production costs and energy demand with increasing CIC is often described by the experience curve model.^3,59−63^ It is a well-established tool that describes the relationship between increasing experience and decreasing costs for a process or product, and it has already been implemented for DAC.^1,8,9,51,64−66^ With each doubling of the CIC, a cost component decreases by a constant factor known as the learning rate (LR). This concept provides reasonable results for known processes but can be misjudged for emerging technologies.^67^ Therefore, in the present work, costs and energy demand are calculated using the experience curve model for ACL and TVSA, referring to and based on the already published literature. For AEC and ESA, the respective data is calculated by drawing analogies to similar technologies and is not based on LR. In particular, the relative CapEx for AEC and ESA is adopted from redox flow and lithium-ion batteries, respectively (see the Methods section for details).

Fasihi et al.^64^ estimated the capital expenditures (CapEx) and the energy demand for ACL and TVSA for the years 2020, 2030, 2040, and 2050, assuming a certain CIC in these years. According to their experience curve model, the costs for ACL and TVSA reduce up to 268/222, 111/84, 72/53 and 54/38 €/t_CO2_, respectively.^64^ Young et al.^51^ conducted a cost analysis of ACL, AEC, TVSA, and MgO ambient weathering for a first-of-a-kind and N^th^-of-a-kind plant, respectively, with a focus on a location analysis. That included economic parameters such as energy prices, the discount rate, CO_2_ transportation costs, construction costs, and carbon intensities that vary across different locations. They refer to the net removed cost of CO_2_, which includes the CO_2_ emissions over the life cycle. In addition, CO_2_ compression and transport are included to calculate the cost of 251–612 (103–444), 784–1538 (445–1346), 328–1329 (166–634), and 277–780 (102–544) $/t_CO2_ for the respective technologies for a CIC of 1 Mt_CO2_/a (1 Gt_CO2_/a) for a case in the USA paired to nuclear electricity and a heat pump for TVSA. In their analysis, the combustion of natural gas is required to achieve high temperatures for ACL and MgO ambient weathering. The cost range is mainly determined by applying different capital cost LRs of 5–19%. The LRs for the operational cost are chosen to be between 0% and 5%. The data for ACL is used from Keith et al.,^13^ AEC from Sabatino et al.,^54^ MgO looping from McQueen et al.,^68^ and the data for TVSA is calculated within their work.^51^ They concluded that the LCOC for AEC in all of their considered scenarios was not lower than the LCOC for the other three processes.

## Methods

Four technologies are considered in the present work:

(1) Absorption with regeneration *via* chemical looping (ACL) as described by Keith et al.^13^ (TRL 7)
(2) Absorption with electrochemical regeneration (AEC); as a reference process, we chose carbonate regeneration using a bipolar membrane described by Iizuka et al.^53^ (TRL 2–3)
(3) Temperature-vacuum-swing adsorption (TVSA) with the reference process described by Wurzbacher et al.^69^ (TRL 8)
(4) Electro-swing adsorption (ESA) as described by Voskian and Hatton^35^ (TRL 1)

ACL and TVSA are nonelectrochemical processes, AEC and ESA can be seen as their respective counterparts with electrochemical steps. All four technologies deliver a high-purity CO_2_ stream. Compression, transport, storage, or utilization of CO_2_ are excluded from our consideration, as they are independent of the technologies used for DAC. The technology readiness level (TRL) for every technology is determined following the framework proposed by Buchner et al.^70,71^ For technologies (1) and (3), material and energy balances and data on capital expenditure (CapEx) are adopted from the literature. For ACL and TVSA, we applied the experience curve model to estimate future costs. Data for the electrochemical devices of processes (2) and (4) are not yet available to the required extent to perform an experience curve model. Instead, optimistic cases/lower limits and pessimistic cases/upper limits are applied to include uncertainties. The CapEx is estimated by assuming analogies: the regeneration cell of the AEC process is assumed to have the same relative CapEx as redox flow batteries, and the electrochemical cell of the ESA process is assumed to have the same relative CapEx as lithium-ion batteries. Details of the data sources and assumptions for the individual technologies are given below.

The costs are assessed in a consistent framework, including common boundary conditions (e.g., energy prices and CIC) and consistent costs for commonly used equipment. Consistency is also achieved by excluding the use of natural gas and waste heat. The experience curve model is implemented to capture varying maturity and future development of ACL and TVSA. The net LCOC is introduced to include the influence of greenhouse gas emissions caused by electricity production.

### Experience Curve Model

The data for energy demand and CapEx gathered from the literature are valid only for the reported development state of the technology. Here, we measure this development state with the CIC *p* of the device/process. Given some reported relative cost or energy demand *c*_initial_ and CIC *p*_initial_, the cost/energy demand *c* for further CICs is calculated by using the experience curve model as follows:
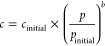
1

Therein, *b* is the experience rate, which is related to the LR defined as the fractional reduction in unit cost per doubling of the CIC:
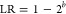
2

In the present work, the learning rates are applied individually to the CapEx and energy demand of the contactor and regeneration step of ACL and TVSA. Representing the cost of a system by adding the individual components with individual LRs usually leads to more proper costs as a holistic consideration of the process.^72^ Since a wide range of LRs and resulting LCOC is assumed, a more detailed breakdown of the components would provide no added value as the resulting LCOC for different LRs of different components would still be in the range of LCOC reported in the present work. The division into contactor and regeneration is necessary to transfer the contactor data from ACL and TVSA to the electrochemical counterpart processes.

### General Cost Model

In the present work, the LCOC values of ACL, TVSA, AEC, and ESA are calculated for best/optimistic and worst/pessimistic initial data and LRs, respectively. Extensive analysis of already published data is performed to create the respective ranges of initial data and LRs: the range is based on data published by the Research Agenda of the National Academy of Sciences^6^ and published by Keith et al.^13^ Further data found in publications is within this range and/or related to the same literature sources.^28,41,51,64^ The collected initial data for ACL and TVSA are given in Table 1, including the CapEx and energy demand. The LRs are also varied in the largest range found in publications.^51,64^ For CapEx, LRs of 5–15% and for the energy demand LRs of 1–5% are applied in this work. These learning rates include the cost effects of individual plant size: we assume that future plants will be built at the most cost-efficient capacity. For AEC and ESA, the cost and energy demand for the contactor are adapted from ACL and TVSA, respectively. The electrochemical processing units are calculated as reported below.

**Table 1:** Parameters for the Experience Curve Model Calculating the Capital Expenditure (CapEx), Specific Electrical Energy Demand *w*_el_, and Specific Heat Demand *w*_heat_ for Components of the Technologies Absorption *via* Chemical Looping (ACL) and Temperature-Vacuum Swing Adsorption (TVSA)^a^

	*C*_CapEx,initial_, M$		energy demand, MWh/t_CO_2__			
			minimum		maximum	
	minimum	maximum	*w*_el_	*w*_heat_	*w*_el_	*w*_heat_
ACL: contactor	210	420	0.24		0.32	
ACL: regeneration	465	835		1.46		3.52
TVSA: contactor	46.7	178	0.16		1.08	
TVSA: regeneration	36.5	143		0.95		5.36

a The initial absolute CapEx *C*_CapEx,initial_ refers to an initial cumulative installed capacity and plant size of 1 Mt_CO_2__/a. Data is adopted from Keith et al.^13^ and from the Research Agenda of the National Academy of Sciences.^6^

For CapEx calculation, we only consider the fixed capital investment (FCI) because of the small TRL of the technologies, which is adopted from the literature and scaled with the experience curve model for ACL and TVSA. (For AEC and ESA, optimistic and pessimistic values of relative CapEx are adopted from analogous technologies instead of assuming an LR). The working capital is not included in the calculations, as the small TRL allows only a rough assessment of the CapEx. From the input data and the experience curve model, we obtain the relative cost *c*_CapEx_ by relating the absolute CapEx *C*_CapEx_ to the plant capacity *p*_plant_.
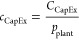
3

The absolute CapEx consists of the investment cost for the process units. Sorbent costs are assumed to be covered by OpEx.^29^ Note that the plant’s capacity is irrelevant for relative measures like the LCOC for our calculations. The economy of scale is assumed to be included in the learning rate if applicable.

The annualized CapEx *C*_CapEx,an_ is calculated assuming a constant interest rate *r* and a given number of payments *x*, which is equal to the economic lifetime of the plant in years (assumed to be equal to the payback period).
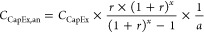
4

For the results in this work, we assumed an economic lifetime of 20 years and an effective annual interest rate of 7%.

The annualized operational expenditure (OpEx) *C*_OpEx,an_ is composed of the annual costs for maintenance and operation *C*_M&O,an_, annual energy costs *C*_energy,an_, and annual sorbent replacement costs *C*_sorb,an_.
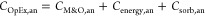
5

A factor *f*_M&O_ of *C*_CapEx_ calculates the *C*_M&O,an_.
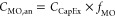
6

For the present work, the factor is chosen to be 3.3%/a. The specific heat demand is converted to specific electrical energy demand *w*_el_ in MWh/t_CO2_ (for calculation, see below) to calculate the energy costs *C*_energy,an_ as follows:
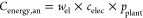
7

where *c*_elec_ is the electricity price in $/MWh. We generally assume 8000 h of operation per year. The annual sorbent (replacement) cost *C*_sorb,an_ is calculated according to eq 8.
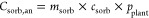
8

The required sorbent masses to capture one ton of CO_2_*m*_sorb_ are further discussed below. The sorbent production cost *c*_sorb_ is calculated for each sorbent and CIC used in the present work (see the Supporting Information for details).

The total annualized cost *C*_total,an_ is calculated as follows:
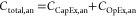
9

The LCOC *c*_LCOC_ per ton of CO_2_ is calculated as follows:
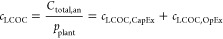
10where *c*_LCOC,CapEx_ is the portion of the CapEx and *c*_LCOC,OpEx_ is the portion of the OpEx on the LCOC.

When carbon intensities of electricity *e*_GG_ are included, the net LCOC is calculated according to
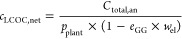
11

The DAC process is carbon-negative when the product *e*_GG_*w_el_ is smaller than 1. This means that the DAC process must capture more CO_2_ than is emitted during production of the consumed electricity. The results in the present work are given for two CICs (1 Mt_CO2_/a, 1 Gt_CO2_/a), respectively. To convert € into US$, both currencies are considered of equal value (1 US$ = 1 €). All results are given in $ of the year 2019; if needed, the source material data was corrected to $2019 using the chemical engineering plant cost index (CEPCI).^73^

### Provision of Heat

In order to compare the technologies, we assume that all of the energy input is electrical energy. Heat is provided by acoustical heat pumps. The coefficient of performance (COP) of the heat pump is calculated according to eq 12, where *T*_amb_ is the ambient temperature on the inlet and is assumed to be 293 K. *T*_req_ is the required temperature for the DAC process on the outlet of 373 K for TVSA^28^ and 1173 K for ACL,^13^ respectively. The exergy efficiency η_exergy_ is assumed to be 75% as a typical heat pump efficiency.^74^ Based on these assumptions, a COP of 3.5 and 1 are obtained, respectively, which is in good agreement with the literature.^75,76^ The use of a heat pump is therefore considered only for TVSA.
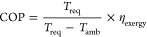
12

### Process-Specific Data and Operations

Flow sheets for the four studied technologies are given in the Supporting Information.

#### Absorption with Chemical Looping (ACL)

The absorption plant with regeneration *via* chemical looping is separated into the air contactor and modules needed for regeneration of the CO_2_-loaded sorbent like the slaker, causticizer, and calciner (see Keith et al.^13^ and Figure S1 for details). Electrical energy is needed to operate the fans, and heat is also required during regeneration.

#### Absorption with Electrochemical Regeneration (AEC)

The absorption plant with electrochemical regeneration is divided into the air contactor and electrochemical regeneration unit (see Iizuka et al.^53^ and Figure S2 for details). The energy demand of the electrochemical step *w*_el,AEC_ is assumed to be only dependent on the voltage *U* and the specific charge *q*:
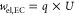
13
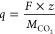
14where *F* is the Faraday constant (96,485 C/mol), *z* = 1 is the number of transferred electrons, and *M*_CO_2__ the molecular mass of CO_2_. The relative CapEx of the electrochemical unit *c*_AEC_ is assumed to be identical to the relative CapEx of a redox flow battery and amounts to between 200 and 3300 $/kW.^77^ The investment cost *C*_CapEx,AEC_ of the regeneration unit are obtained by eq 15.
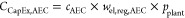
15

### Temperature-Vacuum-Swing Adsorption (TVSA)

The TVSA capture plant includes the contactor (blower and contactor), the regeneration unit (vacuum pump and condenser), a heat pump, and sorbent material (see Deutz and Bardow,^28^Figure S3, and Table S2 for details). The capital cost of heat pump *C*_CapEx,HP_ is calculated using eq 16.
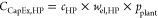
16

The relative capital cost of the heat pump *c*_HP_ amounts to 1090 $/MW,^78^ and the energy demand of the heat pump *w*_el,HP_ is taken equal to the energy demand for regeneration. For the present work, a range of adsorbent costs and properties is adapted from the Research Agenda of the National Academy of Sciences^6^ (see Table 2). The required sorbent mass per ton of CO_2_*m*_sorb,TVSA_ is calculated according to
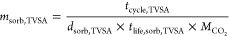
17where *t*_cycle,TVSA_ is the cycle time, *t*_life,sorb,TVSA_ is the lifetime, and *d*_sorb_ is the adsorption capacity of the TVSA sorbent.

**Table 2:** Range of CO_2_ Capture Capacity, Cycle Time, Lifetime, and Purchase Cost of Sorbent Materials for TVSA^6^

property	minimum	maximum
cycle time, s	960	2520
Lifetime, a	0.5	5.0
capacity, mol_CO_2__/kg	1.0	1.5
purchase cost, $/kg	15	50

### Electro-Swing Adsorption (ESA)

The ESA plant consists of a blower providing the air stream and the ESA cell (see Voskian and Hatton^35^ and Figure S4 for details). Electrical energy is needed to operate the blower and the ESA cell. The energy demand of the electrochemical step *w*_el,ESA_ is assumed to be only dependent on the voltage *U* and the charge *q* (see eqs 13 and 14). A range for the capital cost is calculated. The relative CapEx of the electrochemical unit *c*_ESA_ is taken identical to the relative CapEx of a lithium-ion battery and amounts between 600 and 3500 $/kW.^77^ The investment cost *C*_CapEx,ESA_ of the electrochemical unit are obtained by eq 15.

## Results

### Energy Demand

**Figure 4:**
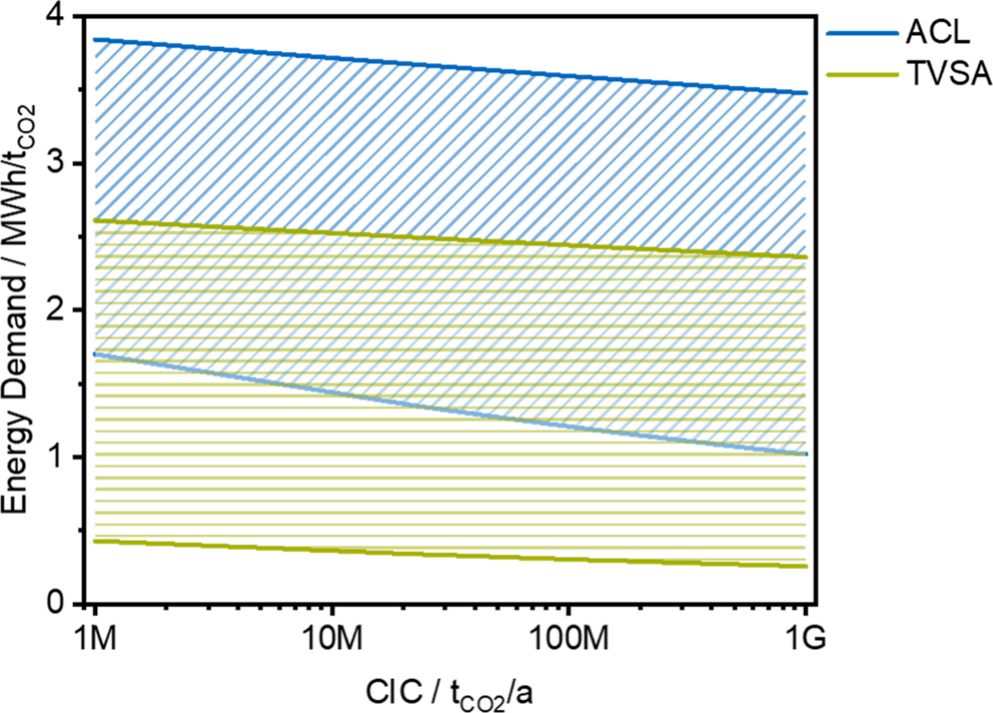
Overall energy demand obtained for absorption *via* chemical looping (ACL) and temperature-vacuum swing adsorption (TVSA) as a function of the cumulative installed capacity (CIC). The hatched area covers the range of initial values and the respective development over the CIC for LRs between 1 and 5%.

When comparing different technologies, the energy demand is more meaningful than the energy costs, as it is independent of external parameters, such as the electricity price. Figure 4 compares the overall energy demand obtained for ACL and TVSA dependent on the CIC and based on various initial values. The hatched area covers the range of initial values and the respective development over the CIC for LRs between 1 and 5%. For both technologies in Figure 4, there is great uncertainty in the energy demand of more than 2 MWh/t_CO2_. Overall, the energy demand of ACL is higher than that of TVSA.

**Figure 5:**
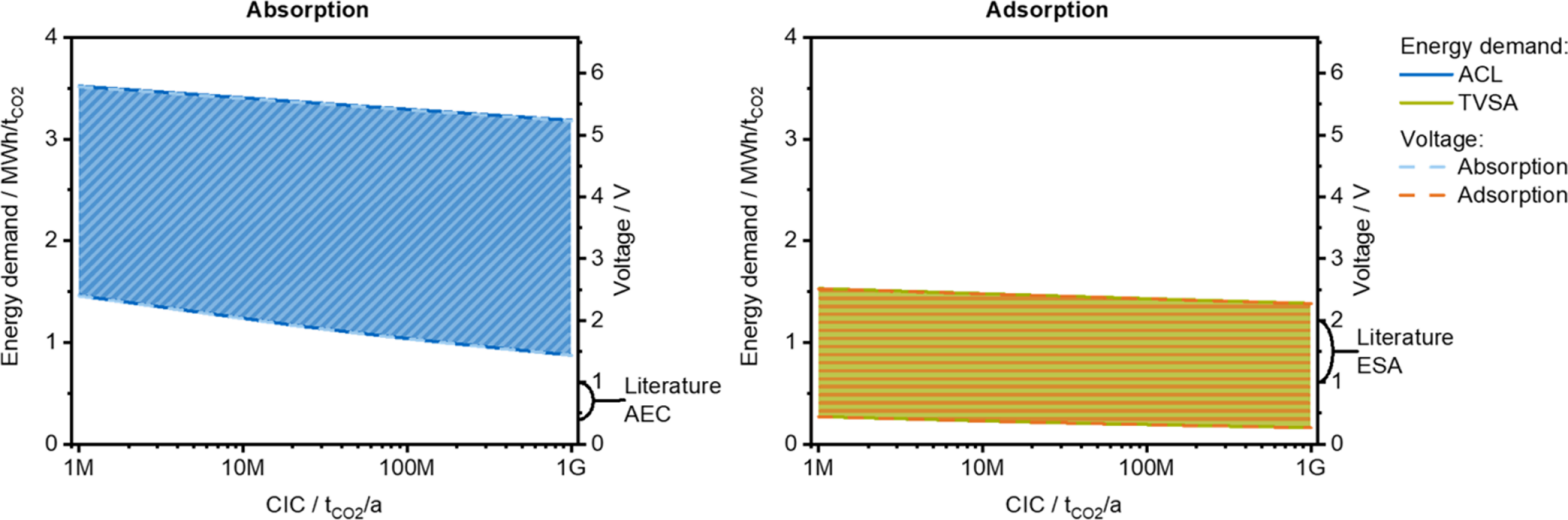
Energy demand needed for regeneration for absorption *via* chemical looping (ACL) and temperature-vacuum swing adsorption (TVSA) as a function of the cumulative installed capacity is shown on the left axis. The energy demand is converted to equivalent cell voltages (right axis), and the number of transferred electrons amounts to 1. The current voltages reported in publications for absorption with electrochemical regeneration (AEC) and electro-swing adsorption (ESA) are given in brackets on the right axis. The dashed area covers the range of initial values and the respective development over the CIC for LRs between 1 and 5%.

Figure 5 shows the energy demand needed for regeneration for ACL (Absorption) and TVSA (Adsorption) on the left axis. The energy demand for operating the fans/contactor is not included, as it is independent of regeneration. When comparing energy demand between Figures 4 and 5, it is noticeable that the energy requirement for the contactor for TVSA is up to 0.9 MWh/t_CO2_, while it is smaller than 0.2 MWh/t_CO2_ for ACL. Regarding the feasibility of the values in Figure 5, it has to be noted that the reaction enthalpy for the release of CO_2_ from CaCO_3_ of 1130 kWh/t_CO2._^13^ For the optimistic edge, the LR approach (with LR > 3%) predicts reaching that limit. This energy demand will only be feasible if heat can be provided by heat integration,^79,80^ which is challenging for high-temperature heat of 900 °C, or the reaction system is changed. For TVSA, the heat of adsorption is the decisive factor determining the minimum energy demand. Depending on the sorbent material and its properties, the respective heat of adsorption is between 0.25 and 0.57 MWh/t_CO2_.^6,31,33,81−84^ It should be noted that a COP of 3.5 of the heat pump is included in the energy demand of the regeneration step; the low energy demand for regeneration for TVSA in Figure 5 is therefore plausible.

In short, the thermodynamic minimum energy demand for ACL and TVSA is related to the reaction enthalpies or the heat of adsorption. Redox potentials, corresponding to voltages, determine the thermodynamic minimum energy demand for electrochemical reactions. Thus, when electrochemical steps replace thermal regeneration, the respective energy demands need to be expressed in terms of cell voltages. In Figure 5, the energy demand required for regeneration for ACL and TVSA is converted to cell voltages on the right axis. The voltages in Figure 5 should be seen as equivalent cell voltages needed for the electrochemical steps for AEC and ESA to achieve an energy similar to or lower than that of ACL and TVSA and give an estimation about the respective limits but do not reflect actual cell voltages of the processes. Note that the voltages in Figure 5 are ideal cell voltages, neglecting any efficiencies and losses, and that the number of transferred electrons is 1 for AEC and ESA, which is due to the processes and underlying electrochemical reactions listed in most comparable processes.^23−27,35−37,39^ Still, other electrochemical reactions with different numbers of transferred electrons could change the cell voltages (according to eq 14). The voltage range for absorption with 1.4–5.4 V is broader than for adsorption with 0.7–2.3 V in Figure 5 at the gigaton scale, indicating that voltages above 5.4 V for AEC and 2.3 V for ESA should be seen as insufficient in the long term. Current publications report voltages of 0.4–1.0 V for AEC^24−27^ and 1.0–2.0 V for ESA,^36−38^ which are given as a reference in brackets on the right axis in Figure 5. For AEC, these voltages are lower than the equivalent voltage range and within the range for ESA. The voltages reported in current publications refer to current laboratory setups under defined conditions and are partly measured at higher CO_2_ concentrations than 400 ppm. The voltages could increase, e.g., due to upscaling effects or atmospheric CO_2_ concentrations but could also decrease for increasing CICs due to optimization of the processes. Overall, this comparison shows that there is a fair chance, especially for absorption, that the energy requirement of the electrochemical DAC processes can be lower than that of nonelectrochemical DAC processes.

A high energy demand also increases the net LCOC, where energy-related CO_2_ emissions are included. Thus, decreasing energy demand has two positive effects on the capture cost: lower energy costs as part of the OpEx and less influence of the carbon intensity on the net LCOC. It is also possible to determine whether a DAC process is carbon-negative for a particular value of the carbon intensity of electricity. For average carbon intensities of photovoltaic (57 kg_CO2e_/MWh^85^) and wind power (18 kg_CO2e_/MWh^85^), ACL, AEC, TVSA, and ESA are carbon-negative over the whole range of the CIC considered in the present work. Even when the maximum carbon intensity of electricity reported in 2022 worldwide is assumed, which amounts to 800 kg_CO2e_/MWh,^86^ all four DAC technologies would be carbon-negative at a CIC of 1 Gt_CO2_/a for the lower limit of the energy demand range (for values of the net LCOC, see Table S5).

### Levelized Cost of Capture (LCOC)

**Figure 6:**
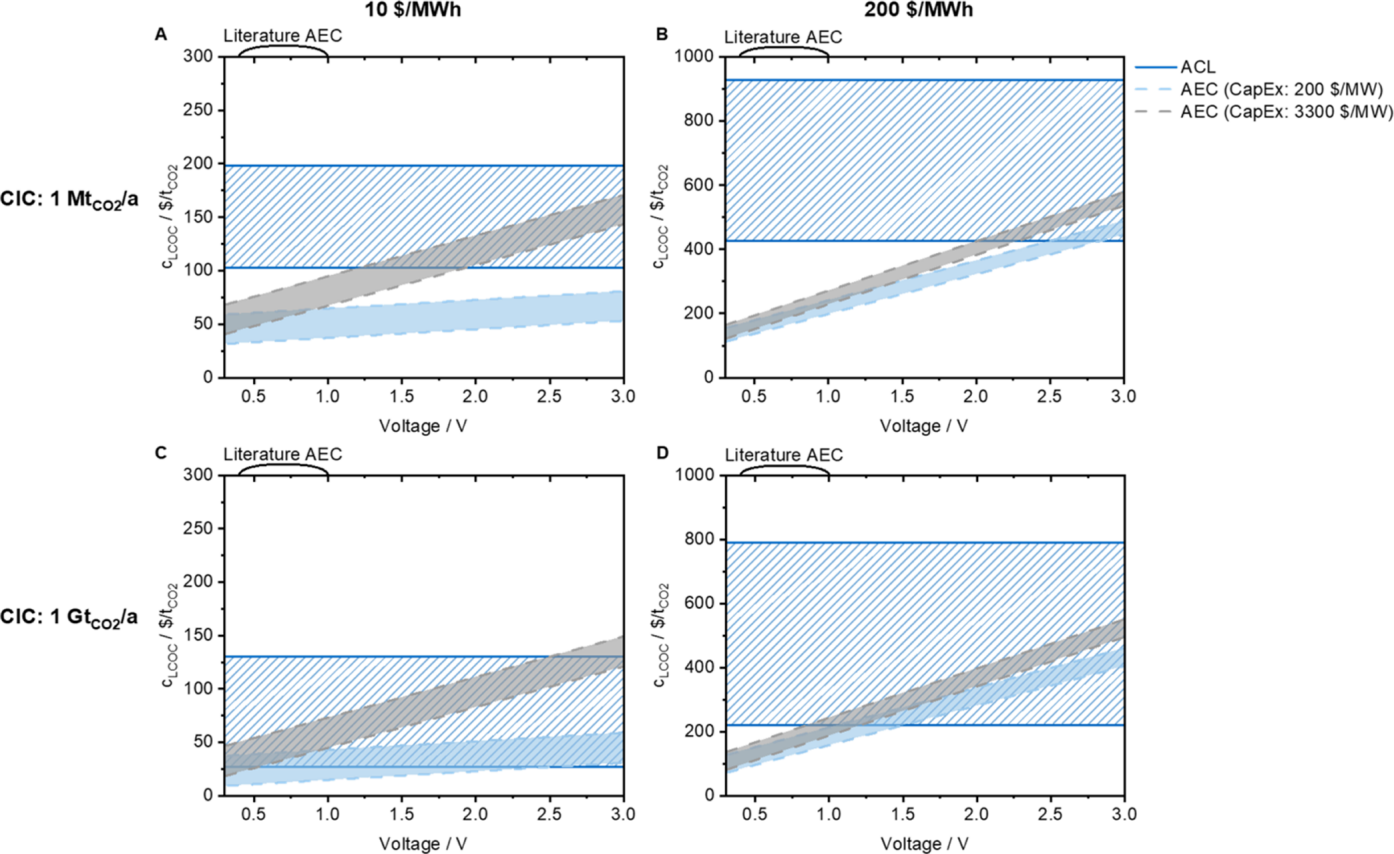
Levelized cost of capture (LCOC) for absorption *via* chemical looping (ACL) and absorption with electrochemical regeneration (AEC) is given dependent on the voltage for a cumulative installed capacity of 1 Mt_CO_2__/a (A, B) and 1 Gt_CO_2__/a (C, D), electricity prices *c*_elec_ of 10 $/MWh (A, C) and 200 $/MWh (B, D), respectively. The capital cost of the electrochemical components amounts to 200 $/MW (light blue)/3300 $/MWh (gray) for AEC.

**Figure 7:**
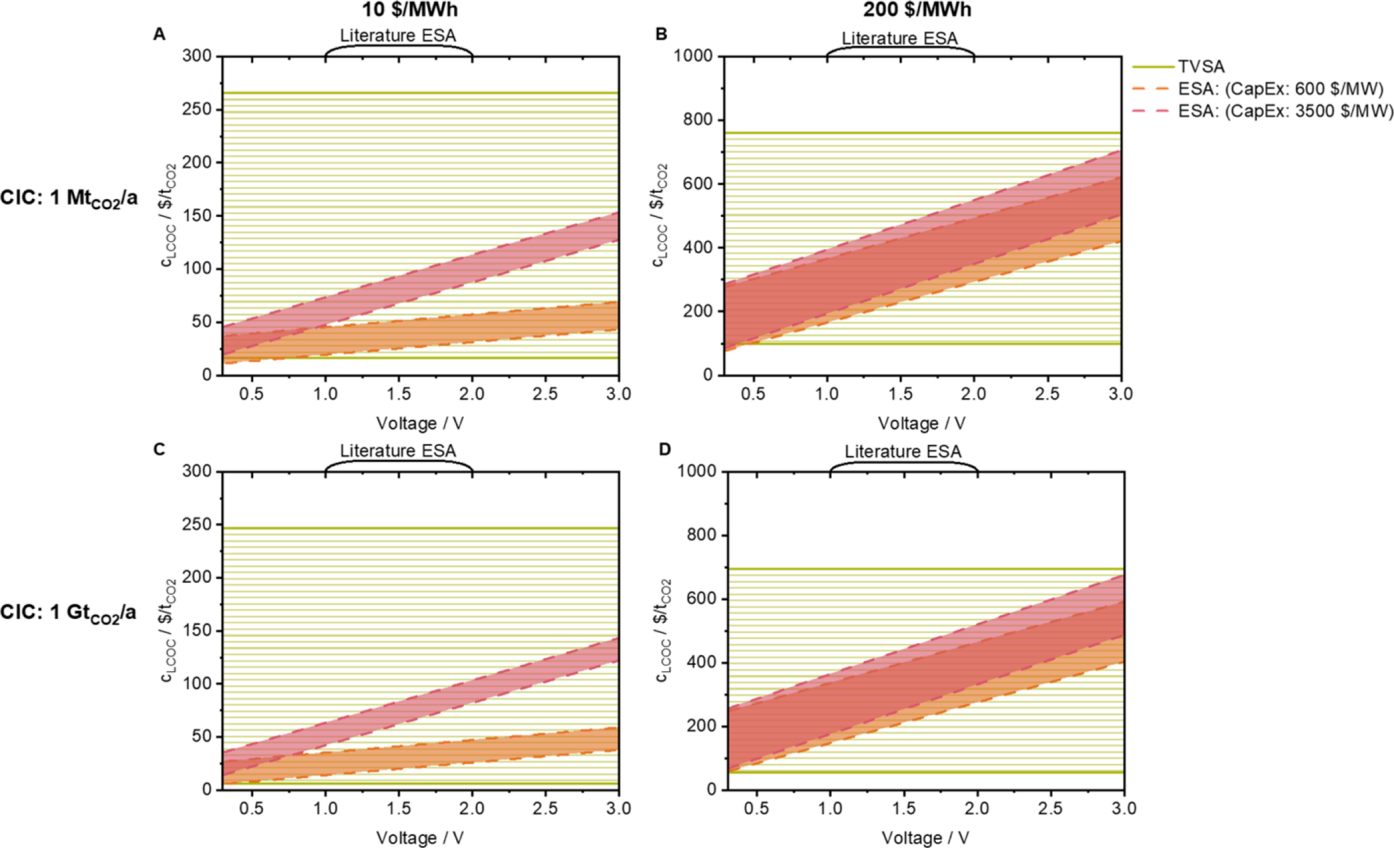
Levelized cost of capture (LCOC) for temperature-vacuum swing adsorption (TVSA) and electro-swing adsorption (ESA) is given dependent on the voltage for a cumulative installed capacity of 1 Mt_CO_2__/a (A, B) and 1 Gt_CO_2__/a (C, D), electricity prices *c*_elec_ of 10 $/MWh (A, C) and 200 $/MWh (B, D), respectively. The capital cost of the electrochemical components amounts to $600/MW (orange)/$3500/MW (red) for ESA.

For the remainder of the present work, the LCOC, and not the net LCOC, is considered. The LCOC for ACL, AEC, TVSA, and ESA is given in Figures 6A–D and 7A–D over the voltage for constant CIC, for constant electricity prices of 10 $/MWh (A and C) and 200 $/MWh (B and D), and for different relative capital costs of the electrochemical components (AEC: 200/3300 $/MW, ESA: 600/3500 $/MW). The LCOC of ACL and TVSA is constant in Figures 6 and 7 as it is independent of voltages. The CapEx of the electrochemical-specific components depends on the membrane cost, membrane area, stack cost, and number of stacks. These factors depend on the energy demand and voltage of the process. As introduced earlier, the relative capital costs are adopted from analogous technologies, redox flow, and lithium-ion batteries, and do not include LRs. As for Figure 5, the currently reported voltages of AEC and ESA are given in brackets. The width of the range for the electrochemical DAC processes is determined by the uncertainty of the costs of the contactor. The higher the electricity price, the greater the uncertainty and, thus, the width of the range. In addition, the influence of the CapEx on the LCOC decreases as the electricity price increases: the range of AEC for cheaper CapEx is below ACL and intersects with ACL for costly CapEx above 1.2 V in Figure 6A, while the ranges are closer together in Figure 6B (cross ACL section above 2.0 and 2.5 V, respectively). The range of TVSA is broader than that of ACL, and besides the uncertainty of CapEx and OpEx, there is also sorbent uncertainty. The LCOC of ESA is within the range of TVSA in Figure 7A,B, except for voltages below 0.5 V the LCOC of ESA is below that of TVSA. The LCOC of ACL and TVSA decreases according to the LR in Figures 6 and 7 for a higher CIC. The LCOC of the electrochemical DAC processes decreases less because the LR is only applied to the costs of the contactor (which is equal to ACL and TVSA, respectively). As a result, the LCOC of AEC overlaps with the LCOC of ACL in Figure 6C. Beyond 2.6 V (lower CapEx) and 0.6 V (higher CapEx), the LCOC of AEC is within the range of ACL. In 6D, the LCOC of AEC is below the LCOC of ACL for voltages below 0.8 and 1.0 V, respectively. In the case of adsorption, the qualitative course is similar to Figure 7A,B, with the difference that the LCOC of ESA is below that of TVSA for voltages around 0.4 and 0.1 V (for lower and higher CapEx, respectively). Overall, the LCOC values of AEC and ESA are similar and mainly differ according to the cost of the contactor. An advantage of electrochemical steps can mainly be derived for absorption to reduce cost, whereas due to the almost complete overlap of TVSA and ESA, no clear advantage of electrochemical steps can be currently seen for adsorption. In addition, the technical implementation of electrochemical adsorption is currently more challenging than electrochemical absorption, which is also reflected by the currently higher reported voltages of the ESA. An LCOC below 100 $/t_CO2_, often referred to as a price limit by policymakers, can only be achieved for very cheap electricity prices. The LCOC differs by a multiple when the electricity price is changed. This indicates that statements about the LCOC or carbon removal costs without telling the electricity cost are less meaningful.

**Figure 8:**
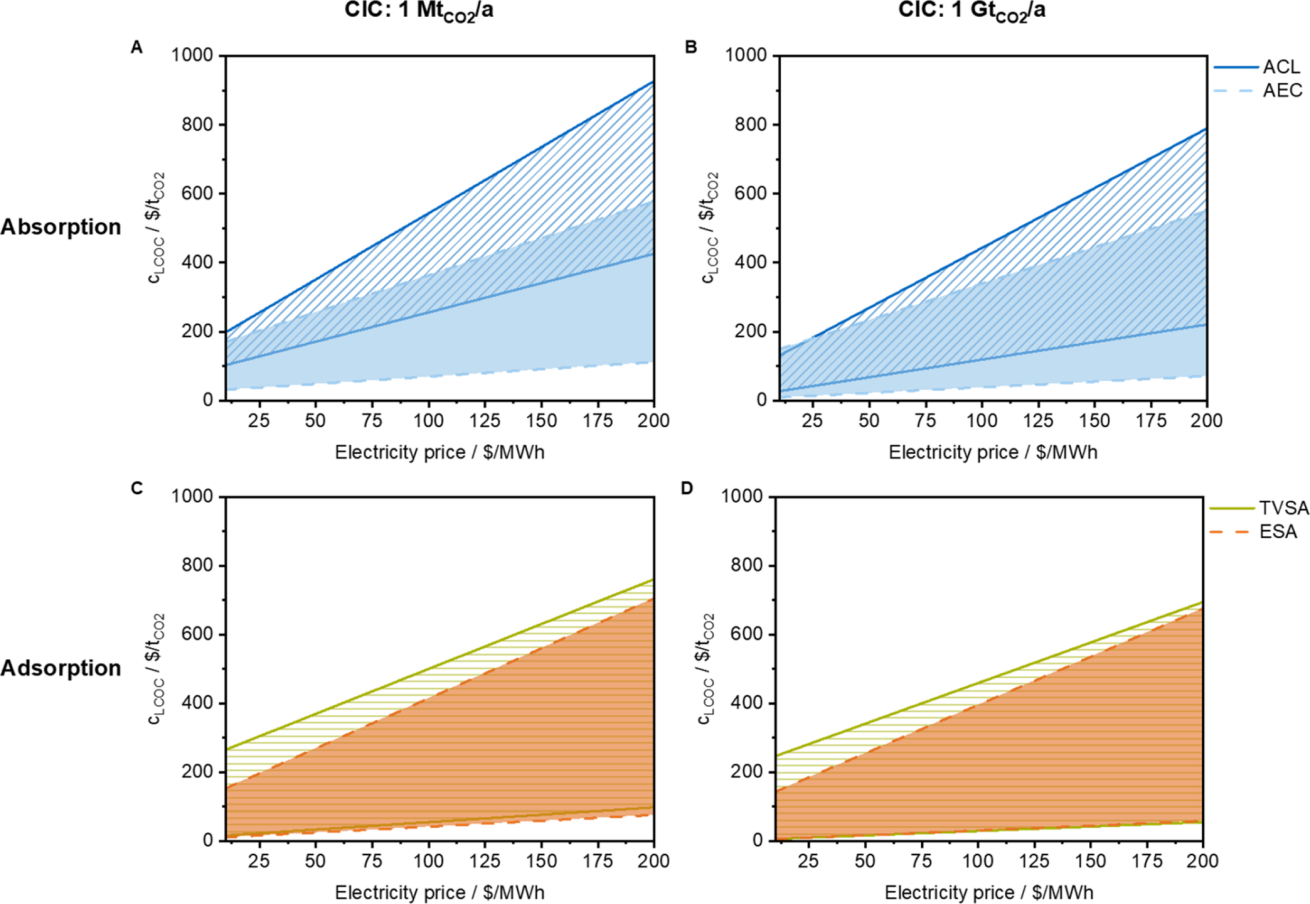
Levelized cost of capture (LCOC) for absorption *via* chemical looping (ACL, blue), temperature-vacuum swing adsorption (TVSA, green), absorption with electrochemical regeneration (AEC, light blue), and electro-swing adsorption (ESA, orange) is given dependent on the electricity price for cumulative installed capacities of 1 Mt_CO_2__/a (A, C) and 1 Gt_CO_2__/a (B, D), respectively. The bottom line is based on a minimum voltage of 0.3 V, capital cost (AEC: $200/MW, ESA: $600/MW), and minimum sorbent costs for TVSA. The top line is based on a maximum voltage of 3.0 V, capital cost (AEC: 3300/MW, ESA: $3500/MW), and maximum sorbent costs for TVSA.

Due to the importance of the electricity price, the LCOC of each technology is plotted dependent on the electricity price in Figure 8 for a CIC of 1 Mt_CO2_/a and 1 Gt_CO2_/a, respectively. The lower limit of the range is based on a minimum voltage of 0.3 V, minimum capital cost (AEC: 200 $/MW, ESA: 600 $/MW) for electrochemical DAC processes, and minimum sorbent cost for TVSA. The top line is based on a maximum voltage of 3.0 V, maximum capital cost (AEC: 3300 $/MW, ESA: 3500 $/MW) for electrochemical DAC processes, and maximum sorbent cost for TVSA. In Figure 8A, the LCOC ranges of AEC and ACL intersect for the upper range of AEC. In Figure 8B, this overlap proportion increases, yet the lower limit of AEC is below that of ACL. Consequently, an advantage of electrochemical regeneration in DAC absorption processes can be observed regardless of the electricity price, with the advantage becoming more pronounced as electricity prices rise. Concerning adsorption, the LCOC range of ESA is within the range of TVSA for both CICs. As a result, no clear advantage of electrochemical steps for the DAC adsorption processes can be found.

## Discussion

Absorption typically incurs higher reaction enthalpies than adsorption, necessitating an increased energy input. For nonelectrochemical DAC absorption processes, the energy is typically provided in the form of (high temperature) heat, where heat integration and waste heat application are limited. In the respective benchmark system of the present work, temperatures of up to 900 °C are needed.^13^ Thus, there is ample space for improvements, either by changing the chemical system to decrease the temperature requirement or by including electrochemical steps. By this, an alternative source of energy input is offered, and the LCOC can be decreased compared to ACL, as shown in the present work. In contrast to absorption, thermal adsorption can benefit from utilizing cheap or waste heat and the low heat of adsorption. Furthermore, the electrochemical step in adsorption faces challenges, in particular, due to chemical instability and decomposition of the sorbent material caused by oxygen reactions.^30,35^ Concerning the sorbent, a constant adsorption/absorption capacity is assumed until the sorbent is replaced. In real applications, the sorbent capacity decreases over time.^87^ This assumption impacts the LCOC, especially in DAC processes with high sorbent costs.

Costs related to water loss are not included in the analysis. These costs vary significantly depending on ambient temperature and humidity,^88^ providing a general statement is therefore not possible. For example, water loss in absorption ranges from 2.6 to 22.8 t_H2O_/t_CO2_ at 20 °C, with humidity ranging from 10% to 90% (see the Supporting Information for calculation). Process water costs are below 40 ct/m^3^^89^ in most regions, leading to an additional cost for water loss cost of 1–9 $/t_CO2_. For adsorption, methods like water harvesting^90,91^ are suggested to separately capture water and CO_2_, thus reducing water loss. In addition, water loss can be limited by the strategic choice of DAC technology and location, as suggested by Küng et al.^92^ Rosa et al.^93^ calculated the water loss of DAC to below 10 m^3^/t_CO2_, which is stated to be significantly lower than that of other carbon capture technologies. In summary, water loss is not expected to significantly increase the LCOC.

When DAC processes are used to achieve negative CO_2_ emissions, an extensive life cycle assessment should be performed to obtain a broad overview of the environmental impacts of the respective DAC processes. For this purpose, the processes must be specified in more detail, e.g., regarding the sorbent material, energy demand, and material of the components. The life cycle assessment by Deutz and Bardow^28^ could serve as a respective example. This is beyond the scope of the present work, where a variety and high uncertainty of DAC processes is considered.

## Conclusions

The range for the energy demand of ACL and TVSA was converted to equivalent cell voltages of 1.4–5.4 V for absorption and to 0.7–2.3 V for adsorption at the gigaton scale to estimate limits for the electrochemical steps in AEC and ESA to achieve a similar or lower energy than ACL and TVSA. Since current literature reports voltages between 0.5 and 1.0 V (AEC) and 1.0–2.0 V (ESA), electrochemical steps in DAC are indeed promising. Not only regarding energy demand but also regarding the LCOC, electrochemical steps are worthwhile studying under the assumption that the cost of the cells is similar to the cost of redox flow (AEC) and lithium-ion batteries (ESA). The LCOC was determined as a function of the voltage, indicating an advantage of electrochemical steps for DAC by absorption. In contrast, no clear advantage of the electrochemical steps in DAC adsorption could be deduced. Furthermore, the LCOC of ACL, TVSA, AEC, and ESA was calculated depending on the electricity price for a voltage range of 0.3–3.0 V for AEC and ESA. Again, electrochemical regeneration in absorption was found to be beneficial compared to thermal regeneration, especially for voltages below 1.8 V, while no clear advantage of an electrochemical step in DAC adsorption could be derived. The LCOC was shown to be significantly dependent on the electricity price, indicating that statements about the LCOC or carbon removal costs without telling the electricity cost are less meaningful. Overall, the present work indicates that an LCOC below 100 $/t_CO2_ can only be achieved for extremely cheap electricity prices around 10 $/MWh. In addition, all four DAC technologies were found to be carbon-negative in operation, independent of the type of electricity.

## Supporting Information Available

Additional information on process design of DAC plants, including flow sheets and sorbent consumption, water loss calculation, sources and values given in Figure 3, and values for the net LCOC (PDF)
