# Supplementary material for: Assessment of the Potential of Electrochemical Steps in Direct Air Capture through Techno-Economic Analysis
Source: Energy Fuels. 2024 Aug 6;38(16):15469–81. doi: 10.1021/acs.energyfuels.4c02202 (PMC11331561; doi:10.1021/acs.energyfuels.4c02202)
Supplement: Supplementary file 1 — ef4c02202_si_001.pdf [file ef4c02202_si_001.pdf]

Supplementary Information

Assessment of the potential of electrochemical steps in direct air capture through techno-economic analysis

Natalie Rosen, [a] Andreas Welter, [b] Martin Schwankl, [b] Nicolas Plumeré, [c] Júnior Staudt, [a] and Jakob Burger[*a]

a. Laboratory of Chemical Process Engineering, Technical University of Munich, Campus Straubing for Biotechnology and Sustainability, 94315 Straubing, Germany.

b. BMW Group, 85748 Garching, Germany.

c. Professorship for Electrobiotechnology, Technical University of Munich, Campus Straubing for Biotechnology and Sustainability, 94315 Straubing, Germany.

***burger@tum.de**

Absorption *via* chemical looping (ACL)

Figure S1 shows the flowsheet of the ACL plant. The sorbent replacement $m_{\mathrm{sorb,OpEx}}$ per ton of CO_2 is adopted from Keith et al..[1] For calculation of c_{sorb}, sorbent raw material costs for lab-scale application are taken from Sigma-Aldrich and converted to large-scale application.[2] As the sorbent is commercially available, no utility, labor, maintenance or synthesis costs must be included. Therefore, the purchase cost equals the overall sorbent cost. Table S1 lists $m_{\mathrm{sorb,OpEx}}$ and c_{sorb} of $CaCO_3$ needed to capture 1 Mt_{CO2}/a. The CO_2 capture rate is 125 t/h.

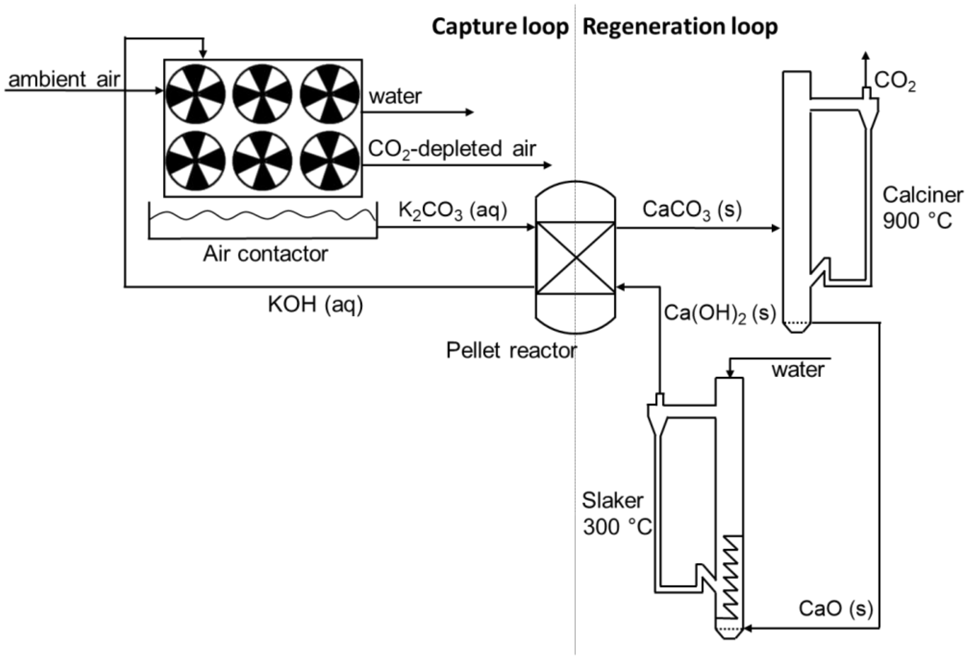

Fig. S1 Flow sheet of the absorption plant with chemical looping.

Table S1: Large-scale raw material quantity and sorbent purchase cost c_{sorb} for ACL sorbent.

**Chemical**	$m_{\mathrm{sorb,OpEx}}$ / t_{sorb}/t_{CO2}	c_{sorb} / $/kg
$CaCO_3$	30[1]	3.12

Absorption with electrochemical regeneration (AEC)

Figure S2 shows the flowsheet of the AEC plant. The sorbent material (costs) are assumed to be part of the CapEx.

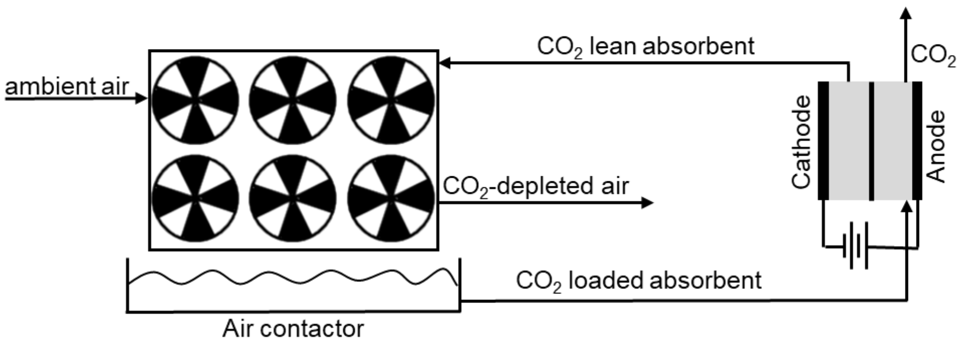

Fig. S2: Flow sheet of the absorption plant with electrochemical regeneration.

Temperature-vacuum swing adsorption (TVSA)

Figure S3 shows the flowsheet of the TVSA plant.

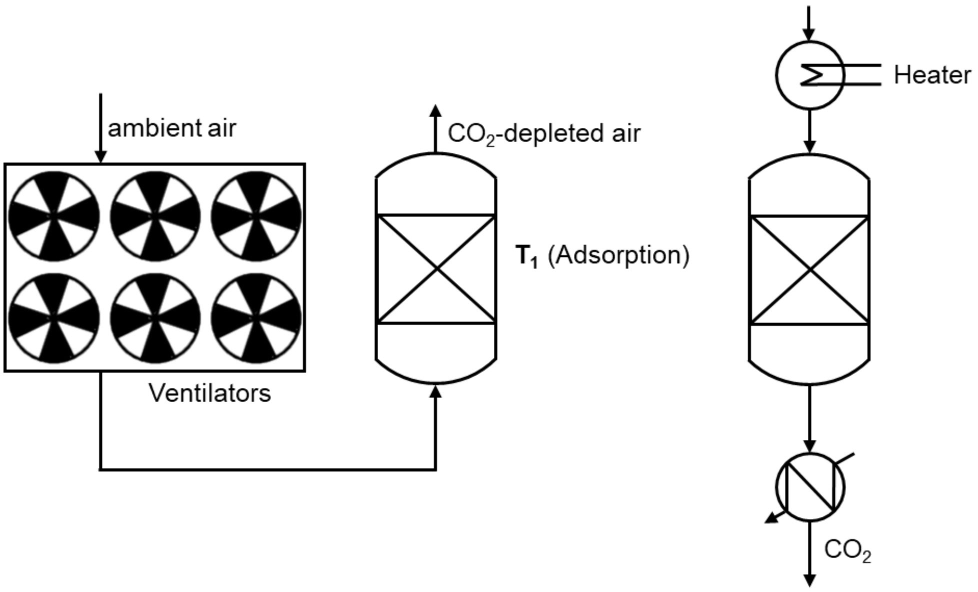

Fig. S3 Flow sheet of the TVSA plant.

A breakdown of capital costs and energy demand for the TVSA plant is listed in Table S2. The values adopted from the National Academy of Sciences (Table 5.10)[3] refer to an annualized payment and were calculated to absolute capital costs using a payback period of 30 years and an interest rate of 12% (according to Equation 4). The energy demand was adopted from Table 5.7[3] and recalculated from GJ/t_{CO2} to MWh/t_{CO2}. Note that implementing a heat pump reduces the initial energy demand by a factor of 3.5, which is the coefficient of performance (COP).

Table S2: Breakdown of capital costs (CapEx) and energy demand of the TVSA plant recalculated from the National Academy of Sciences.[3]

Unit		CapEx / M$	Energy demand / MWh/t_{CO2}
Contactor	Blower	29.0-110.4	
	Contactor	17.7-67.7	
Regeneration	Vacuum pump	36.2-140.2	
	Condenser	0.3-3.2	
Thermal energy			0.95-5.36 (without COP)
			0.27-1.53 (with COP)
Electrical energy	Blower		0.15-1.05
	Vacuum pump		(30.6-253)*10^{-4}

Electro-swing adsorption (ESA)

Figure S4 shows the flowsheet of the ESA plant. The sorbent material (costs) are assumed to be part of the CapEx.

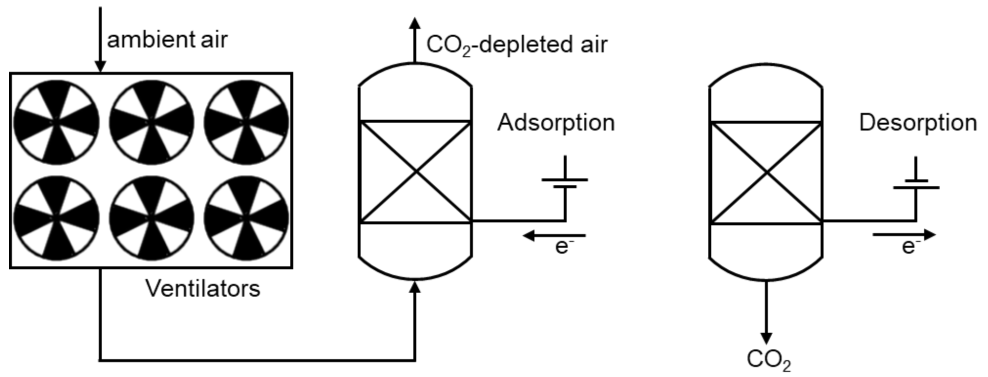

Fig. S4: Flow sheet of the electro-swing adsorption plant.

Calculation of water loss

The relative water content of air $\boldsymbol{x}$ is calculated according to:

$$\boldsymbol{x} = \frac{\boldsymbol{R_a} * \boldsymbol{\varphi} * \boldsymbol{p_s}}{\boldsymbol{R_v} * (\boldsymbol{p_0} - \boldsymbol{\varphi} * \boldsymbol{p_s})}$$

with $\boldsymbol{R_a}$ as the gas constant of air, $\boldsymbol{R_v}$ as the gas constant of vapour, $\boldsymbol{\varphi}$ as the relative humidity, $\boldsymbol{p_s}$ as the saturated pressure pf the vapour at given temperature, and $\boldsymbol{p_0}$ as the overall pressure (ambient pressure). The water loss $\boldsymbol{wl}$ is calculated according to:

$$\boldsymbol{wl} = \boldsymbol{m_a} * (\boldsymbol{x_s} - \boldsymbol{x})$$

where $\boldsymbol{m}_{\mathbf{a}}$ is the amount of air and $\boldsymbol{x}_{\mathbf{s}}$ is the vapour content at saturation. Table S3 lists the respective values at 20 °C.

Table S3: Paramters for water loss calculation at 20 °C.

Parameter	Value
$\boldsymbol{R}_{\mathbf{a}}$	0.2872 kJ/(kg*K)[4]
$\boldsymbol{R}_{\mathbf{v}}$	0.4615 kJ/(kg*K)[4]
$\boldsymbol{p}_{\mathbf{s}}$	23.37 mbar[4]
$\boldsymbol{x}_{\mathbf{s}}$	14.887 g/kg[4]
$\boldsymbol{m}_{\mathbf{a}}$	1695 t

Review of techno-economic studies

Table S4 lists the values and sources that were used to prepare Figure 3 in the main article.

Table S4: The levelized cost of capture (LCOC) for absorption with chemical looping (ACL), absorption with electrochemical regeneration (AEC), and temperature vacuum swing adsorption (TVSA) from different sources.

Source	DAC Technology	Value of LCOC / \$/t_{CO2}
**10.1016/j.joule.2018.05.006** [1]	ACL	94-232
**10.3389/fenrg.2020.00092** [5]	ACL	273-1227
**10.1016/j.oneear.2023.06.004** [6]	ACL	103-444
**10.1016/j.oneear.2023.06.004** [6]	ACL	251-612
**10.1016/j.jclepro.2019.03.086** [7]	ACL	54-268
**10.1002/aic.16607** [8]	TVSA	86-221
**10.1021/acs.iecr.6b03887** [9]	TVSA	60-190
**10.1021/acs.est.0c00476** [10]	TVSA	223
**10.1016/j.oneear.2023.06.004** [6]	TVSA	328-1329
**10.1016/j.oneear.2023.06.004** [6]	TVSA	166-634
**10.1016/j.jclepro.2019.03.086** [7]	TVSA	38-222
**10.1021/acs.iecr.2c00889** [11]	AEC	241-415
**10.1021/acs.iecr.2c00889** [11]	AEC	819-1604
**10.1016/j.oneear.2023.06.004** [6]	AEC	445-1346
**10.1016/j.oneear.2023.06.004** [6]	AEC	784-1539
**10.1016/j.seppur.2012.09.016** [12]	AEC	180
**10.1021/acs.iecr.9b05641** [13]	AEC	773

Net LCOC

The net LCOC is calculated for carbon intensities of 18/57/800 kg_{CO2e}/MWh, an electricity price of 200 $/MWh, and a CIC of 1 Mt_{CO2}/a and 1 Gt_{CO2}/a, respectively, and given in Table S5.

Table S5: The net LCOC for carbon intensities of 18/57/800 kg_{CO2e}/MWh, an electricity price of 200 $/MWh, and a CIC of 1 Mt_{CO2}/a and 1 Gt_{CO2}/a (cn: carbon-neagtive) for the lower and upper limit of the considered range.

**CIC: 1 Mt_{CO2}/a (lower limit)**	**ACL**	**AEC**	**TVSA**	**ESA**
Carbon intensity: 18 kg_{CO2e}/MWh	439.53	98.88	125.34	167.89
Carbon intensity: 57 kg_{CO2e}/MWh	471,80	100,58	127,77	173.15
Carbon intensity: 800 kg_{CO2e}/MWh	not cn	149.57	202.65	430.25
**CIC: 1 Gt_{CO2}/a (lower limit)**				
Carbon intensity: 18 kg_{CO2e}/MWh	225.09	55.46	84.17	149.89
Carbon intensity: 57 kg_{CO2e}/MWh	234.60	56.02	85.47	154.18
Carbon intensity: 800 kg_{CO2e}/MWh	1199.01	69.55	121.14	339.39
**CIC: 1 Mt_{CO2}/a (upper limit)**	**ACL**	**AEC**	**TVSA**	**ESA**
Carbon intensity: 18 kg_{CO2e}/MWh	996,88	799,34	275,92	573,89
Carbon intensity: 57 kg_{CO2e}/MWh	1188,00	894,93	286,47	633,08
Carbon intensity: 800 kg_{CO2e}/MWh	not cn	not cn	1056.43	not cn
**CIC: 1 Gt_{CO2}/a (upper limit)**				
Carbon intensity: 18 kg_{CO2e}/MWh	843.45	726.53	247.80	541.90
Carbon intensity: 57 kg_{CO2e}/MWh	985.94	803.84	256.95	59.93
Carbon intensity: 800 kg_{CO2e}/MWh	not cn	not cn	886.93	not cn

References

(1) Keith, D. W.; Holmes, G.; St. Angelo, D.; Heidel, K. A Process for Capturing CO2 from the Atmosphere. *Joule* **2018**, *2* (8), 1573–1594. DOI: 10.1016/j.joule.2018.05.006.

(2) Hart, P. W.; Sommerfeld, J. T. Cost estimation of specialty chemicals from laboratory-scale prices. *Cost Engineering* **1997** (39), 31–35.

(3) National Academies of Sciences, Engineering and Medicine. Negative Emissions Technologies and Reliable Sequestration: A Research Agenda; National Academy Press, 2019. DOI: 10.17226/25259.

(4) Stephan, K.; Mayinger, F. Thermodynamik:*Grundlagen und technische Anwendungen*, 15., neu bearb. Aufl.; Springer, 2010.

(5) Kiani, A.; Jiang, K.; Feron, P. Techno-Economic Assessment for CO2 Capture From Air Using a Conventional Liquid-Based Absorption Process. *Frontiers in Energy Research* **2020**, *8.* DOI: 10.3389/fenrg.2020.00092.

(6) Young, J.; McQueen, N.; Charalambous, C.; Foteinis, S.; Hawrot, O.; Ojeda, M.; Pilorgé, H.; Andresen, J.; Psarras, P.; Renforth, P.; Garcia, S.; van der Spek, M. The cost of direct air capture and storage can be reduced via strategic deployment but is unlikely to fall below stated cost targets. *One Earth* **2023**, *6* (7), 899–917. DOI: 10.1016/j.oneear.2023.06.004.

(7) Fasihi, M.; Efimova, O.; Breyer, C. Techno-economic assessment of CO2 direct air capture plants. *Journal of Cleaner Production* **2019**, *224*, 957–980. DOI: 10.1016/j.jclepro.2019.03.086.

(8) Sinha, A.; Realff, M. J. A parametric study of the techno-economics of direct CO 2 air capture systems using solid adsorbents. *AIChE Journal* **2019**, *65* (7), 16607. DOI: 10.1002/aic.16607.

(9) Sinha, A.; Darunte, L. A.; Jones, C. W.; Realff, M. J.; Kawajiri, Y. Systems Design and Economic Analysis of Direct Air Capture of CO 2 through Temperature Vacuum Swing Adsorption Using MIL-101(Cr)-PEI-800 and mmen-Mg 2 (dobpdc) MOF Adsorbents. *Industrial & Engineering Chemistry Research* **2017**, *56* (3), 750–764. DOI: 10.1021/acs.iecr.6b03887.

(10) McQueen, N.; Psarras, P.; Pilorgé, H.; Liguori, S.; He, J.; Yuan, M.; Woodall, C. M.; Kian, K.; Pierpoint, L.; Jurewicz, J.; Lucas, J. M.; Jacobson, R.; Deich, N.; Wilcox, J. Cost Analysis of Direct Air Capture and Sequestration Coupled to Low-Carbon Thermal Energy in the United States. *Environmental science & technology* **2020**, *54* (12), 7542–7551. DOI: 10.1021/acs.est.0c00476. Published Online: Jun. 2, 2020.

(11) Sabatino, F.; Gazzani, M.; Gallucci, F.; van Sint Annaland, M. Modeling, Optimization, and Techno-Economic Analysis of Bipolar Membrane Electrodialysis for Direct Air Capture Processes. *Industrial & Engineering Chemistry Research* **2022**, *61* (34), 12668–12679. DOI: 10.1021/acs.iecr.2c00889.

(12) Iizuka, A.; Hashimoto, K.; Nagasawa, H.; Kumagai, K.; Yanagisawa, Y.; Yamasaki, A. Carbon dioxide recovery from carbonate solutions using bipolar membrane electrodialysis. *Separation and Purification Technology* **2012**, *101*, 49–59. DOI: 10.1016/j.seppur.2012.09.016.

(13) Sabatino, F.; Mehta, M.; Grimm, A.; Gazzani, M.; Gallucci, F.; Kramer, G. J.; van Sint Annaland, M. Evaluation of a Direct Air Capture Process Combining Wet Scrubbing and Bipolar Membrane Electrodialysis. *Industrial & Engineering Chemistry Research* **2020**, *59* (15), 7007–7020. DOI: 10.1021/acs.iecr.9b05641.
